# An information theoretic measure of cross-frequency coupling

**DOI:** 10.1186/1471-2202-16-S1-P224

**Published:** 2015-12-18

**Authors:** Silvia C Ardila-Jimenez, Jiaying Tang, Simon R Schultz

**Affiliations:** 1 Centre for Neurotechnology, Imperial College London, London, UK; 2 Department of Bioengineering, Imperial College London, London, UK

## 

The coupling of neuronal oscillations between cortical areas has been proposed both as a mechanism for top-down and bottom-up signaling in the brain. These interactions may facilitate the coordination of both local and distributed networks across different time scales. However, we are still exploring what the best method is to quantify them. Of particular interest has been the role of phase-amplitude cross-frequency coupling (CFC), and a variety of methods to analyze this have been proposed [1]. Thus far none of these stands out as an ideal measure. We propose the use of Mutual Information to quantify CFC. We test the performance of this method against two other approaches: the Mean Vector Length, and the Envelope to Signal Correlation. Finally we apply this method to data recorded from the mouse early visual system.

The ability of Mutual Information to measure the amount of common information shared by the two systems, while capturing both linear and nonlinear relationships, makes it a suitable candidate as a measure of cross-frequency interactions. The resulting value is measured in an absolute scale which provides a framework for comparison across studies. A number of methods have been proposed for measuring CFC each with its own benefits and caveats.

We show that the Mutual Information can quantify phase amplitude cross-frequency interactions on an absolute scale. This method performs at least as well as other CFC measures in identifying the presence of CFC, while not being restricted to linear interactions. Furthermore, it is robust to phase shifts between the low-frequency signal and the amplitude of the high-frequency signal, making it less prone to produce false negative results. Applying the method to experimental data shows apparent CFC interactions between the 1-4Hz band in the LGN and the 10-20Hz and 31-35Hz bands in V1.

**Figure 1:**
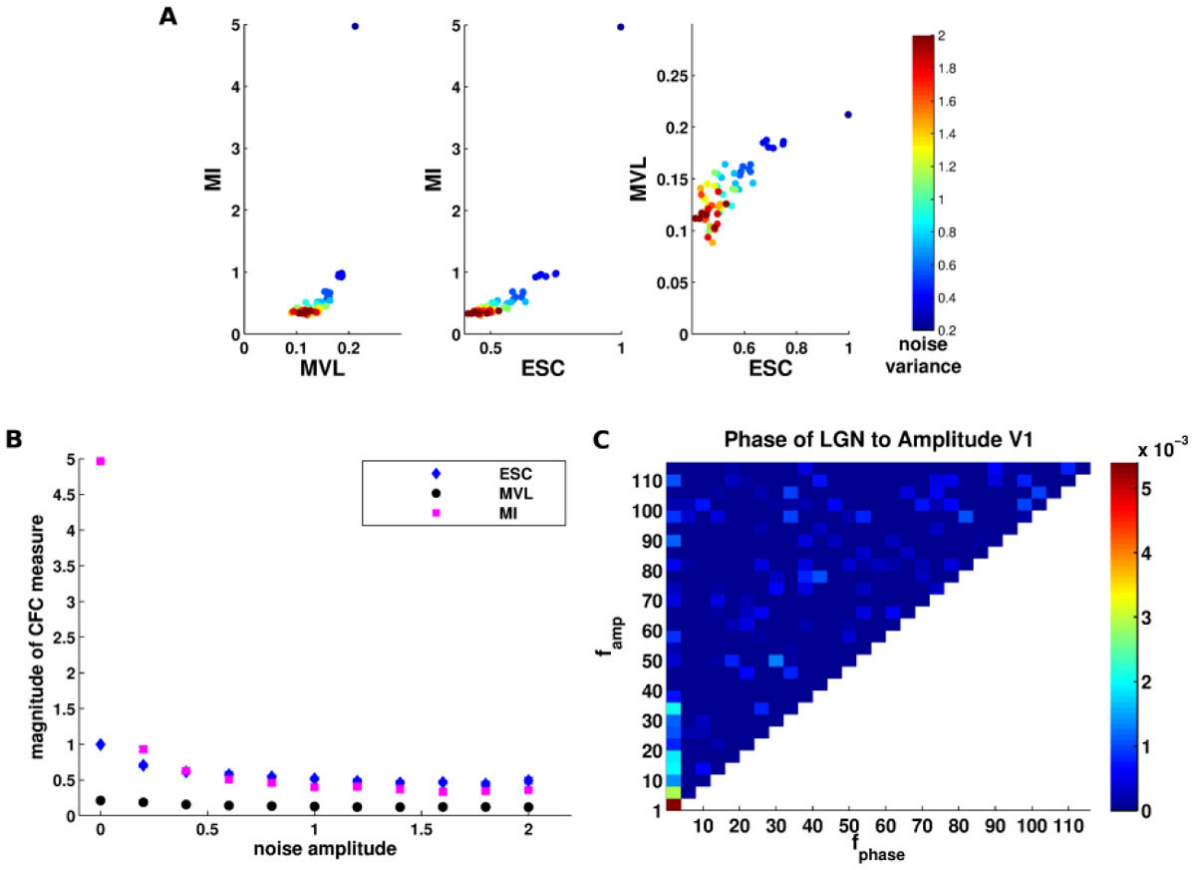
**Mutual Information performance compared against other methods**. **A**. Scatter plots of CFC measures using a synthetic data MVL vs. MI, ESC vs. MI and ESC vs. MVL. Different colors indicate different noise variance. **B**. Mean and variance of the CFC measures for the different levels of noise. **C**. MI used to explore CFC between the thalamus and cortex.
